# Bub1 is required for maintaining cancer stem cells in breast cancer cell lines

**DOI:** 10.1038/srep15993

**Published:** 2015-11-02

**Authors:** Jeong Yoon Han, Yu Kyeong Han, Ga-Young Park, Sung Dae Kim, Joong Sun Kim, Wol Soon Jo, Chang Geun Lee

**Affiliations:** 1 Research Center, Dongnam Institute of Radiological & Medical Sciences, Busan 619-953, ROK; 2 Department of Molecular Biology, College of Natural Sciences, Pusan National University, Busan 609-735, ROK

## Abstract

Breast cancer is a leading cause of death among women worldwide due to therapeutic resistance and cancer recurrence. Cancer stem cells are believed to be responsible for resistance and recurrence. Many efforts to overcome resistance and recurrence by regulating cancer stem cells are ongoing. Bub1 (Budding uninhibited by benzimidazoles 1) is a mitotic checkpoint serine/threonine kinase that plays an important role in chromosome segregation. Bub1 expression is correlated with a poor clinical prognosis in patients with breast cancer. We identified that depleting Bub1 using shRNAs reduces cancer stem cell potential of the MDA-MB-231 breast cancer cell line, resulting in inhibited formation of xenografts in immunocompromised mice. These results suggest that Bub1 may be associated with cancer stem cell potential and could be a target for developing anti-breast cancer stem cell therapies.

Breast cancer is one of the most common cancers and is a leading cause of death among women worldwide due to therapeutic resistance and cancer recurrence^1^,^2^,^3^. Several possible mechanisms have been proposed to explain resistance of cancers, including breast cancer to anti-cancer therapies and cancer recurrence. One is the cancer stem cell (CSC) theory in which small cancer cell subsets, which are capable of self-renewal, become more tumorigenic and resistant to anti-cancer therapeutics than others^4^,^5^,^6^. Thus, many efforts have been devoted to develop CSC-targeted therapies^7^,^8^,^9^,^10^.

Several markers and methods have been suggested to define breast CSCs, such as surface expression of CD24^low^CD44^high^, CXCR4^+^, Hoechst 33342 exclusion activity (side population), and aldehyde dehydrogenase activity^11^,^12^,^13^,^14^,^15^. Among them, CD24/CD44 staining is the most widely used method to identify breast CSCs. CD44, a type I transmembrane glycoprotein, is the major cellular receptor for hyaluronan (HA). Interactions between CD44 and HA are critical for expansion, differentiation, and pluripotency of stem cells including CSCs and also for cancer progression by modulating cell adhesion, migration, and invasion^16^,^17^,^18^,^19^. CD44 cooperates with HA-mediated motility (RHAMM or CD168) to deliver signals from extracellular HA, which is overexpressed in many advanced cancers including breast cancer^20^,^21^,^22^,^23^. RHAMM is an HA cell surface receptor and is also a cytoplasmic and nuclear protein that interacts with interphase microtubules, the mitotic spindle, and centrosomes^24^,^25^,^26^.

Mitotic kinases and mitotic checkpoint proteins play key roles in cell division and maintenance of chromosome stability. Thus, mis-regulation of these processes may result in tumorigenesis. Furthermore, mitosis plays important roles balancing stem cells between self-renewal and differentiation to progenitor cells by regulating symmetric and asymmetric division^27^,^28^. These proteins have been shown to play an important role maintaining the CSC population and could be a potential therapeutic target for CSC-targeted therapies. Plk1 is a key mitotic regulator that has been identified as a potential therapeutic target for eliminating breast and colon CSCs^29^,^30^. CSCs are maintained by Aurora kinase A (AurA) and inhibiting AurA has been viewed as effective approach to target the CSC population in various cancers^31^,^32^,^33^. Glioblastoma initiating cells have lethal kinetochore-microtubule attachment defects that are suppressed by BubR1. Thus, BubR1 may be a therapeutic target for glioblastoma CSCs^34^. Besides the involvement of mitotic kinases and mitotic checkpoint proteins in the maintenance of CSCs, genomic instability and polyploidy, which are induced by defects in mitotic kinases or mitotic checkpoint proteins, contribute to generating cancer-stem-like cells^35^,^36^.

Bub1 is a mitotic checkpoint protein that is overexpressed in breast cancer and other cancers, and its expression correlates with a poor clinical prognosis^37^,^38^,^39^,^40^,^41^,^42^,^43^. We identified that depleting Bub1 in the MDA-MB-231 a breast cancer cell line prevents generation of xenografts in immunocompromised mice due to reduced CSC potential in Bub1-depleted cells and resulted in radiosensitization. Reduced CSC population and radiosensitization by Bub1 depletion were also observed in the MCF7 cells. These results suggest that Bub1, like other mitotic regulators, such as AurA, BubR1, and Plk1, is associated with CSC potential and may be a target for developing of anti-CSC therapy.

## Results

### Depleting Bub1 reduces xenograft forming ability of MDA-MB-231 cells in immunocompromised mice

**Figure 1:**
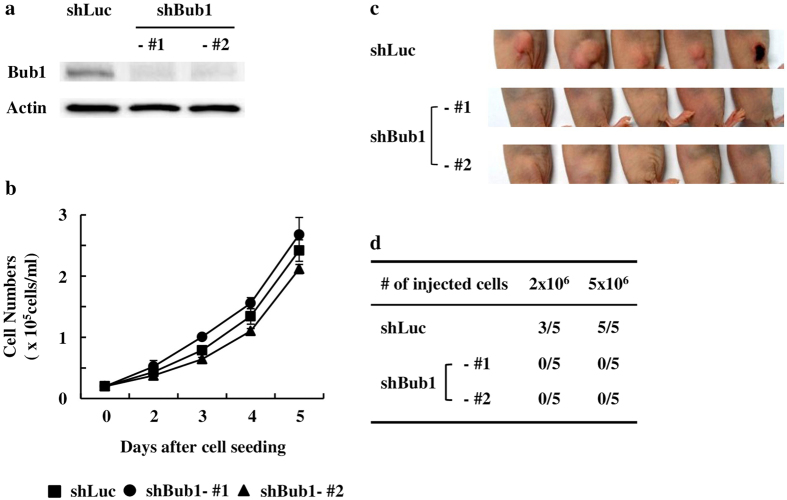
Depleting Bub1 reduces xenograft forming ability of MDA-MB-231 cells in immunocompromised mice. (**a**) The levels of Bub1 expression in the Bub1-depleted MDA-MB-231 stable cell lines were measured by Western blot analysis. (**b**) 2 × 10^3^ cells of each cell lines were seeded onto 6 well plates. Cell numbers were counted at the indicated timpoints. Data were shown as mean values ± SD. (**c,d**) Control (shLuc) and Bub1-depleted (shBub1 #1 & #2) MDA-MB-231 cells were injected subcutaneously into the right flanks of Balb/c-nu mice and allowed to form xenografts. Tumor formation was examined 30 days later.

We used shRNAs targeting Bub1 (shBub1) to investigate the roles of Bub1 in breast cancer development and therapy. After transfection of MDA-MB-231 cells with control shRNA (shLuc), shBub1-#1 or shBub1-#2, stable clones were obtained by puromycin selection. Bub1 depletion in the stable clones was verified by western blot analysis and the reduced expression of Bub1 was maintained during this study (Fig. 1a). Bub1 depletion did not induce significant enhancement or reduction in cell proliferation (Fig. 1b). However, Bub1-depleted MDA-MB-231 cells did not generate xenografts in immunocompromised mice (Fig. 1c,d). Control cells (shLuc) formed xenografts efficiently (8/10 head) but Bub1-depleted MDA-MB-231 cells did not generate xenografts. These results suggest that depleting Bub1 reduces xenograft forming ability of MDA-MB-231 cells in immunocompromised mice.

### Depleting Bub1 reduces the CSC potential of MDA-MB-231 and MCF7 cells

**Figure 2:**
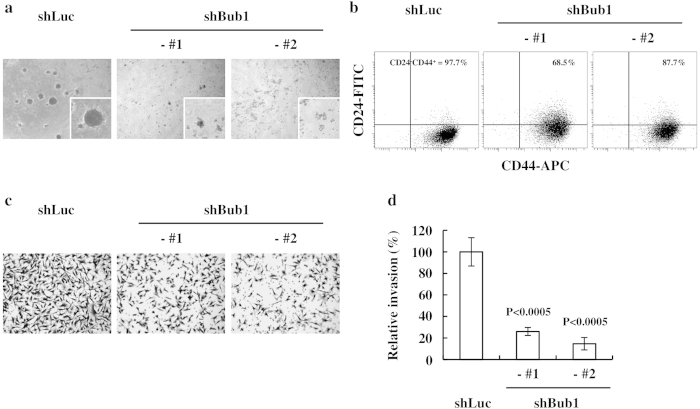
Depleting Bub1 reduces cancer stem cell (CSC) potential of MDA-MB-231 cells. (**a**) Approximately 5 × 10^3^ cells were cultured using a serum-free MammoCult Human Medium kit containing 2.5% Matrigel, and images were obtained 10–14 days later. (**b**) FACS analysis was performed to evaluate the CD24^low^CD44^high^ CSC population. % of CD24^low^CD44^high^ population is shown. (**c,d**) The Matrigel invasion assay was performed, the cells were stained with crystal violet, and images were taken for counting. The number of cells invading the Matrigel was normalized by setting the number of control cells invading the Matrigel to 100%. Data were shown as mean ± Standard Deviation (SD). Statistical significance compared to the control is shown.

**Figure 3:**
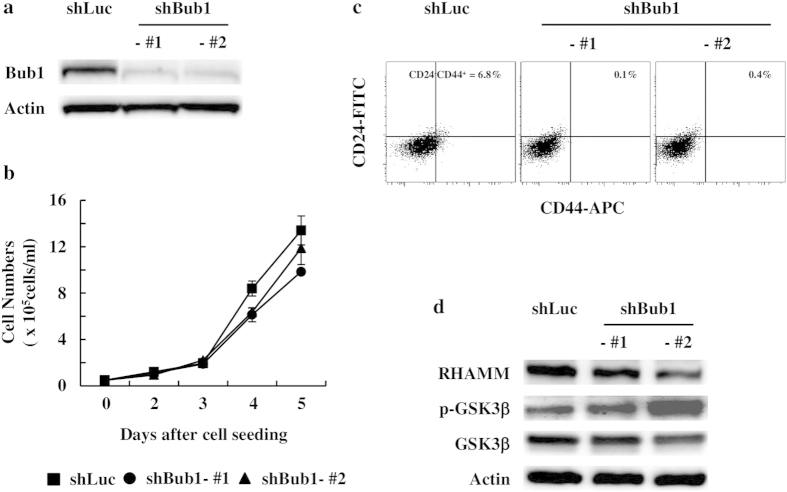
Depleting Bub1 reduces cancer stem cell (CSC) population of MCF7 cells. (**a**) The levels of Bub1 expression in the Bub1-depleted MCF7 stable cell lines were measured by Western blot analysis. (**b**) 5 × 10^3^ cells of each cell lines were seeded onto 6 well plates. Cell numbers were counted at the indicated timpoints. Data were shown as mean values ± SD. (**c**) FACS analysis was performed to evaluate the CD24^low^CD44^high^ CSC population. % of CD24^low^CD44^high^ population is shown. (**d**) Cell lysates of control and Bub1-depleted MCF7 cells were subjected to Western blot analysis using p-GSK3β, GSK3β, and RHAMM antibodies.

A xenograft assay is widely used in immunocompromised mice to evaluate CSC potential. Thus, we investigated whether Bub1-depleted cells had reduced CSC potential. A 3D Matrigel sphere-forming assay was used to evaluate CSC potential. Bub1-depleted cells generated smaller and fewer spheres in 3D Matrigel culture than those in control cells (Fig. 2a). CD24 and CD44 surface staining was used to estimate the CSC population of breast cancer cells. FACS analysis showed that the CD24^low^CD44^high^ population, which is believed to contain breast CSC subsets, was reduced in Bub1-depleted cells compared to that in control cells (Fig. 2b). Enhanced invasion ability is a CSC characteristic. Depleting Bub1 consistently reduced invasion by MDA-MB-231 cells in the Matrigel invasion assay (Fig. 2c,d). Reduced CSC population by Bub1 depletion was further confirmed in MCF7 cells. Bub1 depletion in MCF7 cells did not result in significant reduction in cell proliferation (Fig. 3a,b). Bub1-depleted MCF7 cells were analyzed by FACS analysis using anti-CD24 and CD44 antibodies. Although MCF7 cells have much less CSC population than MDA-MB-231 cells, Bub1 depletion reduced the CD24^low^CD44^high^ population (Fig. 3c). Taken together with the xenograft assay results, these results suggest that depleting Bub1 reduced the CSC potential of breast cancer cell lines.

### Reduced CSC potential correlates with alterations in the RHAMM-GSK3β pathway

**Figure 4 f4:**
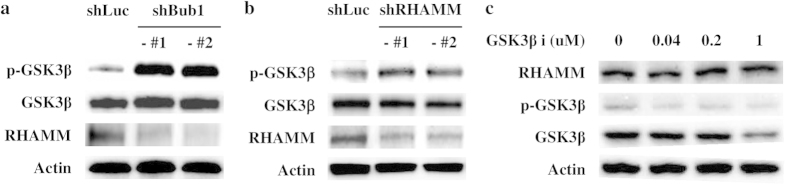
Depleting Bub1 enhances inhibitory phosphorylation of glycogen synthase kinase-3β (GSK3β) through reduced RHAMM expression. (**a**) Cell lysates of control and Bub1-depleted MDA-MB-231 cells were subjected to Western blot analysis using p-GSK3β, GSK3β, and RHAMM antibodies. (**b**) Cell lysates of control and RHAMM-depleted MDA-MB-231 cells were subjected to Western blot analysis using p-GSK3β, GSK3β, and RHAMM antibodies. (**c**) MDA-MB-231 cells were treated with the indicated concentration of a GSK3β inhibitor, and cell lysates were subjected to Western blot analysis using p-GSK3β, GSK3β, and RHAMM antibodies.

We further investigated how depleting Bub1 resulted in reduced CSC potential. RHAMM plays diverse roles on the cell surface and in the nucleus^26^,^44^,^45^. RHAMM influences maintenance of CSCs by regulating GSK3β activity and also is involved in mitotic spindle integrity^45^,^46^. Since Bub1 is a mitotic checkpoint protein involved in regulating the kinetochore-microtubule interaction during mitosis, we investigated whether Bub1 depletion modulate the RHAMM-GSK3β pathway. RHAMM expression was reduced and inhibitory phosphorylation of GSK3β was increased in Bub1-depleted MDA-MB-231 cells compared to those in control cells, indicating that depleting Bub1 may affect the RHAMM-GSK3β signaling pathway (Fig. 4a). These results were further confirmed in Bub1-depleted MCF7 cells (Fig. 3d). In the next step, we used RHAMM shRNAs and a GSK3β inhibitor to investigate the relationship between RHAMM and GSK3β. Depleting RHAMM with shRNAs enhanced GSK3β inhibitory phosphorylation but the GSK3β inhibitor did not modulate RHAMM expression (Fig. 4b,c). These findings suggest that depleting Bub1 decreased RHAMM expression, which, in turn, enhanced inhibitory phosphorylation of GSK3β.[Fig f4]

### Reduced CSC potential by depleting Bub1 results in enhanced sensitivity to irradiation

**Figure 5:**
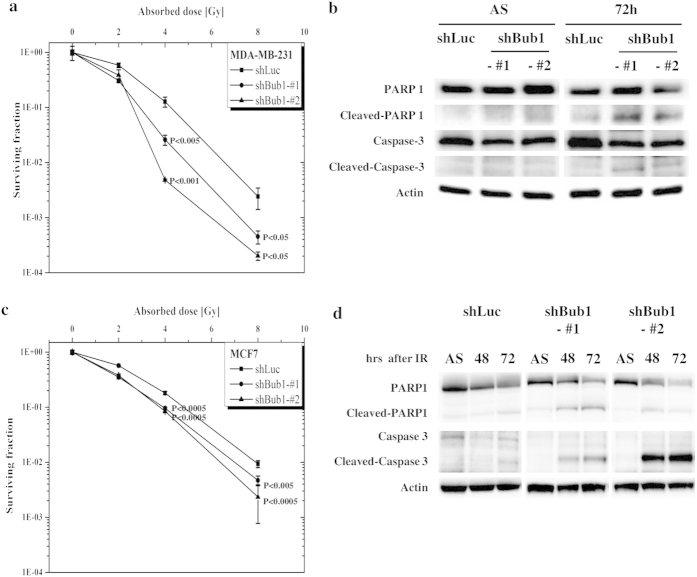
Depleting Bub1 sensitizes breast cancer cells to irradiation. (**a**,**c**) Control and Bub1-depleted MDA-MB-231 or MCF7 cells were irradiated, and clonogenic assays were performed. Data were shown as mean values ± SD. Statistical significance compared to the control is shown. (**b,d**) Control and Bub1-depleted MDA-MB-231 or MCF7 cells were irradiated, and the cells were harvested at the indicated timepoints. Cell lysates were prepared and subjected to Western blot analysis with the indicated antibodies.

CSCs are a main anti-cancer therapy resistance mechanism. Thus, we investigated whether reduced CSC potential of Bub1-depleted MDA-MB-231 or MCF7 cells results in sensitization to anti-cancer therapies. Control and Bub1-depleted MDA-MB-231 or MCF7 cells were compared for their sensitivity to irradiation. Clonogenic assays showed that sensitivity to irradiation was enhanced in Bub1-depleted cells compared to that in control cells (Fig. 5a,c). Enhanced cell death in Bub1-depleted cells by irradiation was further confirmed by enhanced PARP and caspase-3 cleavage (Fig. 5b,d).

## Discussion

CSCs, like stem cells, are maintained through a balance between symmetric and asymmetric division during mitosis. Deregulation of mitotic progression may influence the balance between symmetric and asymmetric division, resulting in changes in the CSC population. This finding suggests that mitotic kinases or mitotic checkpoint proteins may be good targets for developing anti-CSC therapies. AurA, Plk1, and BubR1 are consistently reported to be good targets for developing anti-CSC therapies^29^,^30^,^31^,^32^,^33^,^34^. As a mitotic checkpoint, Bub1 plays important roles in regulating mitosis by interacting with BubR1 and kinetochores. Our results support the possibility that Bub1 can be a target for developing anti-CSC therapies at least in breast cancers, where Bub1 expression correlates with poor clinical prognosis^38^,^39^,^40^,^42^.

Although several mitotic kinases and mitotic checkpoint proteins were reported to be involved in maintaining stem cells or cancer stem cells, specific mechanisms remains poorly understood. Ding *et al.* showed that brain tumor-initiating cells (BTIC) have kinetochore-microtubule (KT-MT) attachment defects and BubR1, specifically GLE2p-binding sequence (GLEBS) domain activity, is required to overcome the defects^34^. In mammary stem/progenitor cell fate determination, it was reported that AurA modulates the balance between the luminal and basal cell lineages by regulating the orientation of the mitotic spindle^47^. These results suggest that it may be important to regulate mitotic microtubules in maintaining stem cell population either in normal organs and cancers.

During mitosis, Bub1 plays several roles, such as recruiting the mitotic checkpoint proteins to the kinetochore, mounting the mitotic checkpoint response and chromosome congression. Bub1 has several protein-protein interaction domains, such as Tetratricopeptide repeat (TPR), GLEBS and a conserved region (CDI) domains involved in recruiting the mitotic checkpoint proteins to the kinetochore and the spindle checkpoint function, and Ser/Thr kinase domain involved in chromosome congression^48^. We need to figure out which domain or activity is involved in modulating CSC population. As described above, GLEBS domain of BubR1 is required to maintain brain tumor-initiating cells (BTIC)^34^. Since Bub1 also has GLEBS domain similar to that of BubR1, it may be interesting to investigate whether GLEBS domain of Bub1 is required to maintain breast cancer stem cells.

RHAMM functions at the cell surface and in the nucleus. Reduced expression of RHAMM is associated with decreased CSC self-renewal and embryonic stem cell pluripotency^46^,^49^. However, it is unclear whether cell surface or nuclear RHAMM is required to maintain self-renewal of stem cells or CSC. Nuclear RHAMM regulates stability of the mitotic spindle, which is involved in chromosome segregation through interactions with kinetochores. As Bub1 is located at the kinetochores during mitosis, depleting Bub1 may influence RHAMM expression by affecting the mitotic spindles and destabilizing RHAMM. Further investigation may be needed to determine how depleting Bub1 affects RHAMM expression and whether cell surface or nuclear RHAMM is important for CSC self-renewal.

As the CSC breast cancer populations are an obstacle to therapeutics, molecules required for CSC maintenance may be promising targets. Our results and previous reports support the possibility that regulation of kinetochore-mitotic microtubule (KT–MT) attachment may be involved in maintaining stem cell or CSC population and can be a target for developing anti-breast CSC therapies.

## Materials and Methods

### Ethics statement

All the methods were carried out in accordance with the approved guidelines at Dongnam Institute of Radiological & Medical Sciences. All experiment protocols were approved by Dongnam Institute of Radiological & Medical Sciences. Animal studies were conducted in accordance with the guidelines established by the Committee on Use and Care of Animals of Dongnam Institute of Radiological & Medical Sciences. Animals were treated humanely in accordance with the Ministry of Food and Drug Safety on the ethical use of animals.

### Plasmid construction, transfection and cell culture

Sense and antisense oligonucleotides for shRNAs targeting human Bub1 (5′-GGAAGTGCCTCATGCTGAAGA-3′, 5′-GCTGCACAACTTGCGTCTACA-3′, 5′-GGGACTGTTGATGCTCCAAAC-3′) were generated, annealed, and cloned into the pSuper.puro vector, according to the manufacturer’s instructions (Oligoengine, Seattle, WA, USA). For Bub1-depleted stable clones, MDA-MB-231 or MCF7 cells were transfected with each shRNA, subject to puromycin selection 36 hrs after transfection. Bub1 expression of stably transfected clones was verified by western blot analysis. shRNAs targeting human RHAMM (TRCN0000061555 and TRCN0000333646) were purchased from Sigma-Aldrich (St. Louis, MO, USA). MDA-MB-231 and MCF7 cells were purchased from the American Type Culture Collection (Manassas, VA, USA). Cell culture, transfection, and irradiation were performed as described previously^50^,^51^.

### Three-dimensional (3D) Matrigel culture, clonogenic assay and quantitative RT-PCR assay

Cells in 3D Matrigel culture were grown in eight-chambered CultureSlides (BD Biosciences, Bedford, MA, USA) at a density of 5,000 viable cells/400 μl using the serum-free MammoCult Human Medium Kit (STEMCELL Technologies, Vancouver, BC, Canada) containing 2.5% Matrigel Matrix (BD Biosciences), according to the manufacturer’s instructions. Images were obtained 10–14 days later. The clonogenic assay was performed as described previously^52^. Surviving fractions were normalized by setting the percentage of surviving colonies in control wells to 1.

### Xenograft assay

Animal studies were conducted in accordance with the guidelines established by the Committee on Use and Care of Animals of Dongnam Institute of Radiological & Medical Sciences. Animals were treated humanely in accordance with the Ministry of Food and Drug Safety on the ethical use of animals. Control and Bub1-depleted MDA-MB-231 cells were inoculated subcutaneously into the right flanks of Balb/c-nu mice (6-weeks-old), and tumor growth was examined.

### Flow cytometry analysis

CD24-FITC and CD44-APC antibodies (BD Pharmingen, San Diego, CA, USA) were used to measure the CD24^low^CD44^high^ CSC-population. Results were analyzed on a Becton Dickinson FACS Aria (Franklin Lakes, NJ, USA) using FACS Diva software.

### Western blot analysis

Protein samples were separated by sodium dodecyl sulfate-polyacrylamide (SDS) gel electrophoresis, transferred to a nitrocellulose filter, blocked, and analyzed with Bub1, poly-ADP ribose polymerase (PARP), cleaved PARP (Santa Cruz Biotechnology, Santa Cruz, CA, USA), RHAMM (Novus Biologicals, Littleton, CO, USA), caspase-3, cleaved caspase-3, p-glycogen synthase kinase 3β (GSK3β) (Cell Signaling Technology, Danvers, MA, USA), and actin (Sigma) antibodies.

### Invasion assay

Cell culture inserts (BD Biosciences) were used to evaluate differences in invasiveness. The upper membranes of the cell culture inserts were coated with a mixture (1:10 ratio) of Matrigel and Opti-MEM^®^I Reduced Serum Medium (Life Technologies, Grand Island, NY, USA) for 1.5 hr in 24-well plates. The cell culture inserts were rehydrated with serum-free DMEM for 1 hr. Approximately 2.5 × 10^4^ cells were suspended in serum-free DMEM and seeded into the upper chambers of the cell culture inserts. DMEM with 10% fetal bovine serum was added to the lower chambers. The cell culture inserts containing cells were incubated at 37°C for 24 hr, and the inserts were removed. The cells that had passed through the membranes were fixed in 100% methanol and stained with 0.1% crystal violet for 10 min. The upper surfaces of the inserts were wiped with a swab, and the membranes were isolated from the inserts to prepare slides. Invading cells were observed and photographed using an Eclipse TS100 inverted microscope (Nikon, Tokyo, Japan) and the Image-Pro Plus program (MediaCybernetics, Bethesda, MD, USA).

### Statistics

Results were expressed as mean ± Standard Deviation (SD). Student’s t-test was used to compare values of test and control samples.

## Additional Information

**How to cite this article**: Yoon Han, J. *et al.* Bub1 is required for maintaining cancer stem cells in breast cancer cell lines. *Sci. Rep.* **5**, 15993; doi: 10.1038/srep15993 (2015).

## References

[b1] Lech R. & Przemyslaw O. Epidemiological models for breast cancer risk estimation. Ginekol Pol 82, 451–454 (2011). 21853936

[b2] Marusyk A. & Polyak K. Tumor heterogeneity: causes and consequences. Biochim Biophys Acta 1805, 105–117, 10.1016/j.bbcan.2009.11.002 (2010). 19931353 PMC2814927

[b3] Merlo L. M., Pepper J. W., Reid B. J. & Maley C. C. Cancer as an evolutionary and ecological process. Nat Rev Cancer 6, 924–935, 10.1038/nrc2013 (2006). 17109012

[b4] O’Brien C. A., Kreso A. & Dick J. E. Cancer stem cells in solid tumors: an overview. Semin Radiat Oncol 19, 71–77, 10.1016/j.semradonc.2008.11.001 (2009). 19249644

[b5] Winquist R. J., Furey B. F. & Boucher D. M. Cancer stem cells as the relevant biomass for drug discovery. Curr Opin Pharmacol 10, 385–390, 10.1016/j.coph.2010.06.008 (2010). 20630801

[b6] Zhou B. B. *et al.* Tumour-initiating cells: challenges and opportunities for anticancer drug discovery. Nat Rev Drug Discov 8, 806–823, 10.1038/nrd2137 (2009). 19794444

[b7] Dean M., Fojo T. & Bates S. Tumour stem cells and drug resistance. Nat Rev Cancer 5, 275–284, 10.1038/nrc1590 (2005). 15803154

[b8] Frank N. Y., Schatton T. & Frank M. H. The therapeutic promise of the cancer stem cell concept. J Clin Invest 120, 41–50, 10.1172/JCI41004 (2010). 20051635 PMC2798700

[b9] Gupta P. B. *et al.* Identification of selective inhibitors of cancer stem cells by high-throughput screening. Cell 138, 645–659, 10.1016/j.cell.2009.06.034 (2009). 19682730 PMC4892125

[b10] Park C. Y., Tseng D. & Weissman I. L. Cancer stem cell-directed therapies: recent data from the laboratory and clinic. Mol Ther 17, 219–230, 10.1038/mt.2008.254 (2009). 19066601 PMC2835048

[b11] Abraham B. K. *et al.* Prevalence of CD44+/CD24−/low cells in breast cancer may not be associated with clinical outcome but may favor distant metastasis. Clin Cancer Res 11, 1154–1159 (2005). 15709183

[b12] Al-Hajj M., Wicha M. S., Benito-Hernandez A., Morrison S. J. & Clarke M. F. Prospective identification of tumorigenic breast cancer cells. Proc Natl Acad Sci USA 100, 3983–3988, 10.1073/pnas.0530291100 (2003). 12629218 PMC153034

[b13] Ponti D., Zaffaroni N., Capelli C. & Daidone M. G. Breast cancer stem cells: an overview. Eur J Cancer 42, 1219–1224, 10.1016/j.ejca.2006.01.031 (2006). 16624548

[b14] Sheridan C. *et al.* CD44+/CD24− breast cancer cells exhibit enhanced invasive properties: an early step necessary for metastasis. Breast Cancer Res 8, R59, 10.1186/bcr1610 (2006). 17062128 PMC1779499

[b15] Shipitsin M. *et al.* Molecular definition of breast tumor heterogeneity. Cancer Cell 11, 259–273, 10.1016/j.ccr.2007.01.013 (2007). 17349583

[b16] Kosaki R., Watanabe K. & Yamaguchi Y. Overproduction of hyaluronan by expression of the hyaluronan synthase Has2 enhances anchorage-independent growth and tumorigenicity. Cancer Res 59, 1141–1145 (1999). 10070975

[b17] Naor D., Nedvetzki S., Golan I., Melnik L. & Faitelson Y. CD44 in cancer. Crit Rev Clin Lab Sci 39, 527–579, 10.1080/10408360290795574 (2002). 12484499

[b18] Stratford A. L. *et al.* Targeting p90 ribosomal S6 kinase eliminates tumor-initiating cells by inactivating Y-box binding protein-1 in triple-negative breast cancers. Stem Cells 30, 1338–1348, 10.1002/stem.1128 (2012). 22674792

[b19] Toole B. P. Hyaluronan promotes the malignant phenotype. Glycobiology 12, 37R–42R (2002). 10.1093/glycob/12.3.37r 11971857

[b20] Adamia S., Maxwell C. A. & Pilarski L. M. Hyaluronan and hyaluronan synthases: potential therapeutic targets in cancer. Curr Drug Targets Cardiovasc Haematol Disord 5, 3–14 (2005). 15720220 10.2174/1568006053005056

[b21] Assmann V., Marshall J. F., Fieber C., Hofmann M. & Hart I. R. The human hyaluronan receptor RHAMM is expressed as an intracellular protein in breast cancer cells. J Cell Sci 111 **(Pt 12)**, 1685–1694 (1998). 9601098 10.1242/jcs.111.12.1685

[b22] Telmer P. G., Tolg C., McCarthy J. B. & Turley E. A. How does a protein with dual mitotic spindle and extracellular matrix receptor functions affect tumor susceptibility and progression? Commun Integr Biol 4, 182–185, 10.4161/cib.4.2.14270 (2011). 21655434 PMC3104573

[b23] Wang C. *et al.* The overexpression of RHAMM, a hyaluronan-binding protein that regulates ras signaling, correlates with overexpression of mitogen-activated protein kinase and is a significant parameter in breast cancer progression. Clin Cancer Res 4, 567–576 (1998). 9533523

[b24] Assmann V., Jenkinson D., Marshall J. F. & Hart I. R. The intracellular hyaluronan receptor RHAMM/IHABP interacts with microtubules and actin filaments. J Cell Sci 112 **(Pt 22)**, 3943–3954 (1999). 10547355 10.1242/jcs.112.22.3943

[b25] Joukov V. *et al.* The BRCA1/BARD1 heterodimer modulates ran-dependent mitotic spindle assembly. Cell 127, 539–552, 10.1016/j.cell.2006.08.053 (2006). 17081976

[b26] Maxwell C. A. *et al.* RHAMM is a centrosomal protein that interacts with dynein and maintains spindle pole stability. Mol Biol Cell 14, 2262–2276, 10.1091/mbc.E02-07-0377 (2003). 12808028 PMC194876

[b27] Chang J. T. & Reiner S. L. Asymmetric division and stem cell renewal without a permanent niche: lessons from lymphocytes. Cold Spring Harb Symp Quant Biol 73, 73–79, 10.1101/sqb.2008.73.008 (2008). 19022740

[b28] Lathia J. D. *et al.* Distribution of CD133 reveals glioma stem cells self-renew through symmetric and asymmetric cell divisions. Cell Death Dis 2, e200, 10.1038/cddis.2011.80 (2011). 21881602 PMC3186899

[b29] Francescangeli F. *et al.* Proliferation state and polo-like kinase1 dependence of tumorigenic colon cancer cells. Stem Cells 30, 1819–1830, 10.1002/stem.1163 (2012). 22753241

[b30] Hu K., Law J. H., Fotovati A. & Dunn S. E. Small interfering RNA library screen identified polo-like kinase-1 (PLK1) as a potential therapeutic target for breast cancer that uniquely eliminates tumor-initiating cells. Breast Cancer Res 14, R22, 10.1186/bcr3107 (2012). 22309939 PMC3496140

[b31] Cammareri P. *et al.* Aurora-a is essential for the tumorigenic capacity and chemoresistance of colorectal cancer stem cells. Cancer Res 70, 4655–4665, 10.1158/0008-5472.CAN-09-3953 (2010). 20460511

[b32] Chefetz I., Holmberg J. C., Alvero A. B., Visintin I. & Mor G. Inhibition of Aurora-A kinase induces cell cycle arrest in epithelial ovarian cancer stem cells by affecting NFkB pathway. Cell Cycle 10, 2206–2214 (2011). 21623171 10.4161/cc.10.13.16348 PMC3154367

[b33] Mannino M., Gomez-Roman N., Hochegger H. & Chalmers A. J. Differential sensitivity of Glioma stem cells to Aurora kinase A inhibitors: implications for stem cell mitosis and centrosome dynamics. Stem Cell Res 13, 135–143, 10.1016/j.scr.2014.05.001 (2014). 24879067 PMC4085484

[b34] Ding Y. *et al.* Cancer-Specific requirement for BUB1B/BUBR1 in human brain tumor isolates and genetically transformed cells. Cancer Discov 3, 198–211, 10.1158/2159-8290.CD-12-0353 (2013). 23154965 PMC3632446

[b35] Liang Y. *et al.* Stem-like cancer cells are inducible by increasing genomic instability in cancer cells. J Biol Chem 285, 4931–4940, 10.1074/jbc.M109.048397 (2010). 20007324 PMC2836097

[b36] Zhang S. *et al.* Generation of cancer stem-like cells through the formation of polyploid giant cancer cells. Oncogene 33, 116–128, 10.1038/onc.2013.96 (2014). 23524583 PMC3844126

[b37] Bie L. *et al.* The accuracy of survival time prediction for patients with glioma is improved by measuring mitotic spindle checkpoint gene expression. PLoS One 6, e25631, 10.1371/journal.pone.0025631 (2011). 22022424 PMC3192043

[b38] Finetti P. *et al.* Sixteen-kinase gene expression identifies luminal breast cancers with poor prognosis. Cancer Res 68, 767–776, 10.1158/0008-5472.CAN-07-5516 (2008). 18245477

[b39] Glinsky G. V., Berezovska O. & Glinskii A. B. Microarray analysis identifies a death-from-cancer signature predicting therapy failure in patients with multiple types of cancer. J Clin Invest 115, 1503–1521, 10.1172/JCI23412 (2005). 15931389 PMC1136989

[b40] Nakagawa T. *et al.* A tissue biomarker panel predicting systemic progression after PSA recurrence post-definitive prostate cancer therapy. PLoS One 3, e2318, 10.1371/journal.pone.0002318 (2008). 18846227 PMC2565588

[b41] Shigeishi H. *et al.* Correlation of human Bub1 expression with tumor-proliferating activity in salivary gland tumors. Oncol Rep 15, 933–938 (2006). 16525682

[b42] Sotiriou C. *et al.* Breast cancer classification and prognosis based on gene expression profiles from a population-based study. Proc Natl Acad Sci USA 100, 10393–10398, 10.1073/pnas.1732912100 (2003). 12917485 PMC193572

[b43] Takagi K. *et al.* BUB1 immunolocalization in breast carcinoma: its nuclear localization as a potent prognostic factor of the patients. Horm Cancer 4, 92–102, 10.1007/s12672-012-0130-x (2013). 23288590 PMC10358026

[b44] Maxwell C. A., McCarthy J. & Turley E. Cell-surface and mitotic-spindle RHAMM: moonlighting or dual oncogenic functions? J Cell Sci 121, 925–932, 10.1242/jcs.022038 (2008). 18354082

[b45] Tolg C. *et al.* RHAMM promotes interphase microtubule instability and mitotic spindle integrity through MEK1/ERK1/2 activity. J Biol Chem 285, 26461–26474, 10.1074/jbc.M110.121491 (2010). 20558733 PMC2924079

[b46] Shigeishi H. *et al.* Maintenance of stem cell self-renewal in head and neck cancers requires actions of GSK3beta influenced by CD44 and RHAMM. Stem Cells 31, 2073–2083, 10.1002/stem.1418 (2013). 23649588

[b47] Regan J. L. *et al.* Aurora A kinase regulates mammary epithelial cell fate by determining mitotic spindle orientation in a Notch-dependent manner. Cell Rep 4, 110–123, 10.1016/j.celrep.2013.05.044 (2013). 23810554

[b48] Elowe S. Bub1 and BubR1: at the interface between chromosome attachment and the spindle checkpoint. Mol Cell Biol 31, 3085–3093, 10.1128/MCB.05326-11 (2011). 21628528 PMC3147602

[b49] Jiang J., Mohan P. & Maxwell C. A. The cytoskeletal protein RHAMM and ERK1/2 activity maintain the pluripotency of murine embryonic stem cells. PLoS One 8, e73548, 10.1371/journal.pone.0073548 (2013). 24019927 PMC3760809

[b50] Han Y. K. *et al.* A possible usage of a CDK4 inhibitor for breast cancer stem cell-targeted therapy. Biochem Biophys Res Commun 430, 1329–1333, 10.1016/j.bbrc.2012.10.119 (2013). 23261434

[b51] Lee C. G. *et al.* Roles of 14-3-3eta in mitotic progression and its potential use as a therapeutic target for cancers. Oncogene 32, 1560–1569, 10.1038/onc.2012.170 (2013). 22562251

[b52] Park G. Y. *et al.* 14-3-3 eta depletion sensitizes glioblastoma cells to irradiation due to enhanced mitotic cell death. Cancer Gene Ther 21, 158–163, 10.1038/cgt.2014.11 (2014). 24626062

