# Corrigendum: Bub1 is required for maintaining cancer stem cells in breast cancer cell lines

**DOI:** 10.1038/srep17984

**Published:** 2016-01-06

**Authors:** Jeong Yoon Han, Yu Kyeong Han, Ga-Young Park, Sung Dae Kim, Joong Sun Kim, Wol Soon Jo, Chang Geun Lee

Scientific Reports 5: Article number: 15993; 10.1038/srep15993published online: 11022015; updated on: 01062016

This Article contains an error in the Acknowledgements section:

“This work was supported by the National Research Foundation of Korea (DIRAMS) grant funded by the Korea government (MSIP) (50597–2014 & 50491–2015). We thank Min Young Kim, and Min Su Ju for administrative support.”

should read:

“This work was supported by the National Research Foundation of Korea (DIRAMS) grant funded by the Korea government (MSIP) (50597–2014). We thank Min Young Kim, and Min Su Ju for administrative support.”

