# Corrigendum: Bub1 is required for maintaining cancer stem cells in breast cancer cell lines

**DOI:** 10.1038/srep33106

**Published:** 2016-11-11

**Authors:** Jeong Yoon Han, Yu Kyeong Han, Ga-Young Park, Sung Dae Kim, Chang Geun Lee

Scientific Reports 5: Article number: 1599310.1038/srep15993; published online: 01 06 2016; updated: 11 11 2016

Joong Sun Kim and Wol Soon Jo did not make contributions that warrant authorship of this paper and have been removed from the author list.

The Acknowledgements section now reads:

This work was supported by the National Research Foundation of Korea (DIRAMS) grant funded by the Korea government (MSIP) (50597-2014).

We thank Min Young Kim, and Min Su Ju for administrative support and J.S. Kim and W.S. Jo for the data analysis and scientific comments.

The Author Contributions statement now reads:

C.G.L. and J.Y.H. designed and wrote the manuscript. J.Y.H., Y.K.H., G.-Y.P. and S.D.K. performed the experiments. All authors reviewed the manuscript.

These errors have now been corrected in the PDF and HTML versions of the Article, as well as the previous Corrigendum that accompanies the Article.

